# How and When Does Psychological Wellbeing Contribute to Proactive Performance? The Role of Social Resources and Job Characteristics

**DOI:** 10.3390/ijerph18052492

**Published:** 2021-03-03

**Authors:** Jean-Sébastien Boudrias, Francesco Montani, Christian Vandenberghe

**Affiliations:** 1 Department of Psychology, Université de Montréal, Montréal, QC H3C 3J7, Canada; jean-sebastien.boudrias@umontreal.ca; 2 Department of Management, Rimini Campus, University of Bologna, 47900 Rimini, Italy; 3 Department of Management, HEC Montréal, Montréal, QC H3T 2A7, Canada; christian.vandenberghe@hec.ca

**Keywords:** psychological wellbeing, proactive performance, leader-member exchange, team-member exchange, job characteristics

## Abstract

Are psychologically healthy employees more proactive at work? Surprisingly, responses to this question are lacking as empirical research has overlooked the wellbeing–proactive performance relationship. Drawing insights from the conservation of resources theory and the motivational fit perspective, this study proposes that leader-member exchange and team-member exchange act as social resources that convey the benefits of psychological wellbeing to subsequent proactive performance. Moreover, job complexity and task interdependence—two job characteristics that enhance the motivational potential of social resources—are expected to amplify these positive indirect relationships. Data from a three-wave, time-lagged study conducted among employees (N = 318) from French-Canadian organizations were used to test our hypothesized model. The results indicated that leader-member exchange mediated a positive relationship between wellbeing and proactive performance and that the contribution of wellbeing to proactive performance via leader-member exchange was increased when job complexity was higher. We also found a negative indirect relationship between wellbeing and proactive performance via team-member exchange when team interdependence was lower. Theoretical and practical implications of this research are discussed.

## 1. Introduction

The relationship between employee wellbeing—a psychological state reflecting a positive experience at work [1,2]—and work performance fascinates organizations [3,4] and researchers [5,6,7,8,9]. Over the years, this relationship has been investigated through various analytical perspectives and operationalizations of performance [9,10]. Yet, surprisingly, prior research has neglected proactive individual performance, a set of self-initiated, future-focused behaviors oriented toward improving work efficacy [11,12]. In this research, we focus on the contribution of psychological wellbeing at work, defined as a domain-specific, psychological gestalt resulting from individuals’ positive evaluations of and affective reactions to their work [9]. Essentially a work-related experience rather than an assessment of one’s physical health [1], this conceptualization captures how people feel psychologically fulfilled at their job [13], as indicated by their overall level of serenity, harmony and involvement feelings.

Examining the psychological wellbeing–proactive performance relationship is an important issue because proactive behaviors represent a sine qua non condition for enhancing individual and organizational effectiveness in today’s uncertain environment [14]. As the successful enactment of proactive behaviors requires energy and a significant amount of resources, psychological wellbeing appears as a major driver that provides the necessary energy enabling the individual to acquire more specific resources supporting proactive behaviors in the face of work challenges [12,15,16]. In that sense, psychological wellbeing can be considered as an energizing resource for proactive performance [17].

The literature linking psychological wellbeing to performance has reported small and variable effects for wellbeing [4,7,9]. This suggests that intervening variables (e.g., mediators and moderators) may come into play for elucidating how and when psychological wellbeing can result in a higher level of proactive performance. Along that line, the current study aims to investigate the mediating role of social exchanges with supervisors and coworkers as mechanisms through which employees who feel well at work access the social resources needed to improve their proactive work performance [18,19].

Furthermore, the wellbeing literature suggests that different situational features can moderate the relationship between employee wellbeing and performance in general [9,10]. However, the scope of these findings is limited because previous research has not addressed the “social processes” through which wellbeing is expected to influence performance. The effect of social resources, such as having developed constructive social exchanges with supervisors or coworkers, would then depend on employees’ job characteristics (e.g., job complexity, social embeddedness/coordination requirements). To the best of our knowledge, such a model (i.e., a moderated mediation model) has been scarcely examined. Exploring such a model would help identify the role of social resources and the associated boundary conditions linking psychological wellbeing to proactive performance. In addition, social processes are considered important in the proactive behavior literature but are controversial [20,21]. Constructive social exchange relationships offer a large pool of resources that help employees engage in proactive endeavors, yet some of these resources (e.g., instrumental or emotional support) may not always be relevant for the accomplishment of one’s job or can come with some restrictive forces for change brought by social cohesion [22,23]. This is a potential explanation for why social exchanges with supervisors and coworkers had weak and inconsistent relations with proactive performance in Cai et al.’s review [20]. The authors suggested that the value of social processes for the enactment of proactive performance is influenced by moderators.

**Figure 1:**
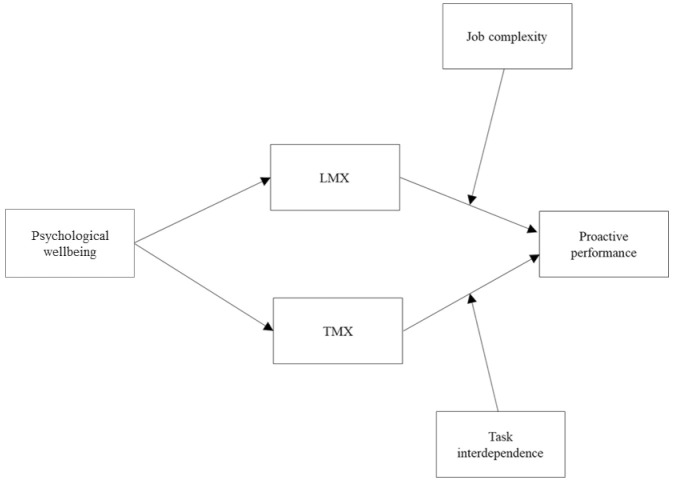
Conceptual model. LMX = leader-member exchange; TMX = team-member exchange.

Taken together, the arguments developed in the wellbeing literature and in the proactive behavior literature highlight the value of integrating social exchange processes and situational characteristics to uncover the mechanisms and boundary conditions that influence the strength of the relationship between psychological wellbeing and proactive performance. However, few theoretical perspectives currently exist to support these claims. Accordingly, to address this important yet overlooked issue, this study integrates two theoretical frameworks, namely the conservation of resources (COR) theory [17] and the motivational fit perspective [24], to propose and test the moderated mediation model of wellbeing and proactive performance (Figure 1). This model posits that psychological wellbeing boosts proactive performance by enabling employees to invest their resources in high-quality social exchanges with supervisors—i.e., leader-member exchange (LMX)—and coworkers—i.e., team-member exchange (TMX). Hence, the relationship between psychological wellbeing and proactive performance would be mediated by LMX and TMX (see Appendix A). Moreover, we propose that the contribution of psychological wellbeing to proactive performance through LMX and TMX would depend on two job characteristics: job complexity and task interdependence, respectively. The development of high-quality social exchange with the leader would be of higher value when the employee has a complex job, while investment in positive exchanges with coworkers would facilitate proactive performance mostly when there is task interdependence among coworkers. 

The present study intends to contribute to the literature in three important ways. First, we examine a more complex perspective of how psychological wellbeing relates to proactive performance (i.e., through mediated moderation relationships) than is previously carried out in the literature. This approach is essential to address the lack of consistency in the strength of the relationship between employee wellbeing and performance as reported in previous studies, and thus provides a more accurate picture of how individuals experiencing wellbeing can achieve their proactive potential. Second, by identifying LMX and TMX as mediators, our study addresses the need for identifying the social exchange mechanisms that connect employee wellbeing to performance [9] and highlight the role of the social pathways through which psychological wellbeing fosters proactive performance [20]. Third, by assessing the moderating role of job complexity and task interdependence, our study extends the wellbeing and proactivity literatures, which have overlooked the conditions that alter the effects of employee wellbeing and social exchange processes on proactive performance [4,20]. This way, our study brings to light that the characteristics of jobs matter in determining the extent to which psychologically healthy individuals involved in high quality social exchanges can behave proactively to improve individual and organizational outcomes.

### 1.1. Mediating Role of Social Exchange Processes: The COR Perspective

Through the lens of the COR theory, psychological wellbeing can be considered as a resource [25]. The main proposition of the COR theory is that individuals strive to protect their resources and make resource investments enabling them to secure valued resources or to gain additional resources [17,26]. As such, wellbeing at work is a valued resource for employees. It is valued for intrinsic reasons (e.g., pleasure) [27] as well as for giving instrumental capacities (e.g., flexibility, openness) to pursue actions in a given context [28,29,30]. According to the COR theory, wellbeing at work is a personal resource that enables resource investments and resource acquisition to optimize one’s adjustment and performance [31]. This process of resource acquisition can be fulfilled through developing functional interpersonal relationships at work. 

Indeed, the COR theory argues that the acquisition of social resources is sought after by people to perform effectively in their work context [32]. The theory proposes that resource acquisition is easier when people already possess a substantial level of resources. Higher levels of resources, such as psychological wellbeing, would place people in a resource gain perspective [32]. Employees with a higher level of wellbeing would be more confident and open with others, interact more frequently with them, and be more inclined to achieve satisfactory social relationships [1,2,4,18]. Feeling serene, socially fitting and involved in their work, psychologically healthy individuals would be better able to see the resources available from others as well as the benefits of further developing a positive social context to continue to obtain resources in the future. This would be achieved because they would be more self-congruent and in line with their work environment [33,34]. In contrast, when people possess fewer resources, they would focus on resource protection instead of resource investment. Individuals experiencing a low level of wellbeing may be susceptible to a resource loss spiral, as they may tend, for instance, to act in a more defensive way with others, to isolate themselves, and to limit their investments to preserve their current (and limited) resources [35,36]. Accordingly, we posit that wellbeing is a personal resource that supports the development of social resources at work, as captured by LMX and TMX constructs.

According to Graen and Uhl-Bien [37], LMX refers to the quality of the relationship between an employee and the supervisor. High LMX is characterized by mutual trust, open exchange of information, and mutual support. Similarly, TMX is defined as the quality of exchanges between an employee and coworkers, including the reciprocal exchange of ideas, honest feedback, and mutual assistance [38]. From a COR theory perspective, LMX and TMX are meaningful social resources because they represent general resources that are useful to work adjustment rather than specific resources to achieve a particular goal. These general resources are relevant when it is not possible to forecast the specific situational requirements or the behaviors that are necessary for meeting such requirements. Proactive performance typically emerges in this type of context [11]. Indeed, the change-related (i.e., uncertain) nature of such behaviors prevents employees from knowing in advance all potential setbacks they could face and the specific resources they need to take proactive initiatives. Employees feeling well at work can invest in the development of these general resources, which could eventually be mobilized to obtain the specific supports needed for enacting their proactive endeavors. This investment strategy is consistent with the COR theory tenets suggesting that individuals orient their efforts to stay well-adjusted to their environment and to leave their behavioral options open to meet the different types of challenges that arise.

The role of social resources in the accomplishment of proactive performance has recently gained attention because “to affect the environment and initiate changes, employees need to seek support from, cooperate with, and build allies with others [20] p. 209”. Indeed, social interactions (e.g., networking) and proactive behaviors are linked [39]. These social interactions can involve the supervisor, coworkers, or more distal organizational representatives. Yet, because supervisors and coworkers are more accessible [40], it is easier for employees to develop exchange relationships with them to constitute ongoing resources. As such, LMX and TMX are general resources reflecting functional vertical (e.g., LMX) and horizontal (e.g., TMX) exchange relationships that can be leveraged for enacting proactive performance. While interactions between LMX and TMX have been found to predict performance in teams [41], our research model assumes that different resources (i.e., that are not completely interchangeable) are available through LMX and TMX. Therefore, these mechanisms are complementary rather than compensatory and their effects are expected to be influenced by specific boundary conditions.

**LMX as Mediator.** Developing positive exchange relationships with one’s supervisor is instrumental to performance [42]. According to meta-analyses, LMX is positively related to various performance outcomes including task performance and citizenship behavior [43,44]. Similarly, Chiaburu et al. [45] found LMX to be positively related to proactive performance. These authors suggested that LMX provides a supportive context that fosters employees’ willingness to be proactive. Indeed, proactive behaviors can be enacted while performing one’s work role creatively [46]. These behaviors, disruptive of the status quo, can be considered as risky as their success is not guaranteed. The supervisor can have a unique role, owing to his or her formal authority, by promoting a supportive context for employees who engage in proactive initiatives. Indeed, supervisors have more power than coworkers to make decisions, reward certain behaviors, and support employee initiatives [47,48]. LMX relationships are therefore important for employees given the required efforts and potential drawbacks entailed in the proactive process. 

Employees need personal resources to be able to figure out how new ways of doing things can be implemented. Studies by Fredrickson [49] indicate that positive feelings, those typically accompanying wellbeing, can facilitate creative thinking. To move from envisioning new ways of doing things to the emission of proactive behaviors, commitment and support from supervisors are crucial [20,50]. Quality relationships with supervisors as fostered by higher employee wellbeing would increase the receptivity of supervisors to proactive behaviors. Employees would feel more secure to propose innovations when they feel the supervisor provides tangible support [51]. High LMX also means that employees may obtain feedback from the supervisor when their initiatives fail and there is a need to take corrective actions [52]. Research by Li et al. [53] indicates that high-quality interactions with supervisors explain why people predisposed to proactivity are more inclined to engage in proactive behaviors. In sum, although LMX does not always have positive implications for performance [22], previous research suggests that LMX can act as a social resource that facilitates the enactment of proactive performance. 

Although LMX has not been considered as a mediator between psychological wellbeing and proactive performance in previous research, indirect support can be found for this relationship. For example, a cross-lagged study [54] indicated that employees’ positive feelings at work facilitated the development of LMX three months later. Further, LMX has been found to mediate the relationship between subordinates’ positive feelings and various performance outcomes [43]. According to Martin et al.’s meta-analysis [42], trust in supervisors’ support is the key mechanism linking LMX to extra-role behaviors such as proactive performance. Thus, the following hypothesis is proposed (Hypothesis 1).

**Hypothesis** **1**  *LMX will mediate a positive relationship between psychological wellbeing and proactive performance.*

**TMX as Mediator.** Horizontal relationships complement vertical relationships in facilitating proactive performance. Coworkers may offer socio-emotional and informational resources that are useful to proactive behaviors [20,55,56]. Indeed, Vough et al. [21] noted that individuals interact with and rely on others in many ways to accomplish their proactive goals, suggesting that interpersonal interactions may facilitate proactive performance. First, exchange relationships with coworkers provide different perspectives that facilitate the generation of new ideas [57], which can fuel the process of envisioning proactive behaviors to improve the work context [50]. Coworkers could further provide a sounding board to test new ideas before voicing them more formally. Getting access to support from coworkers is a key enabler to promote new ideas in a work context, especially if other members of the group are expected to be impacted by the proposed changes [21]. It gives a security base that motivates people to implement their ideas despite obstacles and pursue their efforts even when conflicts arise [23,58]. High-quality relationships with coworkers also lend credibility that proactive behaviors are performed for the common good [59]. Thus, TMX can make coworkers open to proactive behaviors from others in the group. Gong et al.’s study [60] found that the quality of information exchanges and trust in the social environment were key to explain why proactive minded employees displayed proactive behaviors. Therefore, in accordance with the COR theory, many resources can be expected to be developed in a context where psychologically healthy workers develop a high quality TMX. These resources would energize proactive behaviors.

Although TMX has not been studied as a mediator between psychological wellbeing and proactive performance, some studies on social support, where coworkers were involved [35,61], have documented the social mechanisms intervening between wellbeing and performance. Daniels and Guppy [35] found that accountants with higher contentment at work were more likely to receive subsequent help and support. Further, Tsai et al. [61] found in two samples of sales agents that people with a positive mood were more likely to help others and to subsequently receive help from them in return; these positive relationships with coworkers in turn predicted higher persistence in work behaviors and higher performance. Thus, the following hypothesis is proposed (Hypothesis 2).

**Hypothesis** **2**  *LMX will mediate a positive relationship between psychological wellbeing and proactive performance.*

### 1.2. Moderating Role of Job Characteristics: The Motivational Fit Perspective

The motivational fit perspective [24] provides insights that help understand how boundary conditions can alter the contribution of LMX and TMX in the psychological wellbeing–proactive performance relation. This framework suggests that the factors that prompt individuals to engage in a given behavior are unlikely to release their motivational potential unless the situation allows for their expression [62,63]. Cai et al. [20] applied the motivational fit perspective to proactivity and suggested that when the social processes do not fit the characteristics of the job, its influence on proactive behavior is reduced. In support of this idea, a few studies found social processes to interact with job characteristics to predict proactivity. For example, Leung et al. [64] showed that when perceived support for innovation was high, role conflict was positively related to innovative performance. Similarly, Volmer et al. [65] found that high levels of job autonomy enhanced the benefits of LMX for employee involvement in developing new ideas. 

Based on the above premises, we argue that the motivational fit perspective can be integrated with the COR theory to determine the boundary conditions for the wellbeing–social resources–proactive performance relations. Indeed, the COR theory can be viewed as a motivational theory that explains how employees build resources through social investments and, thereby, improve their functioning [25]. The motivational fit perspective would suggest that the relationship between social investments and proactive behavior is stronger when there is a fit between the work situation and the motivational potential underlying social investments. As such, this perspective complements the COR theory by disclosing the boundaries upon which the COR-based predictions on the consequences of the resource building process are most applicable. Job complexity and task interdependence are job characteristics that can align the work situation with the motivational potential of LMX and TMX, respectively, and moderate their effects on proactive performance. We discuss these ideas in the next sections. 

**Job Complexity as a Moderator of LMX**. While LMX is a general asset for work performance, it is particularly relevant in complex jobs, where few generic solutions are available to accomplish tasks involving uncertainty [66,67]. Job complexity and autonomy have both been identified as situational characteristics that can strengthen the link between employee wellbeing and performance [9]. While these characteristics often coexist in the real world, they capture different realities. According to Morgeson and Humphrey’s model [67], complexity is a “knowledge demand” while autonomy is a basic “motivational” characteristic of jobs. We selected the former in our model because it raises the demand for (i.e., reason to) performing proactive behaviors in a job [12]. Further, compared to job autonomy, job complexity is a job requirement that is less dependent on the quality of the relationship with the supervisor [37], making the motivating potential of this job characteristic distinguishable from the motivating potential of LMX.

LMX may be helpful for performing proactive behaviors when tasks are complex, involving intensive knowledge work. In these jobs, proactive behaviors are needed to accomplish work duties because solutions are less readily available [68]. Typically, the extensive knowledge needed in complex jobs means that employee–supervisor relationships must be personalized. Therefore, high-LMX employees may benefit from individualized interactions that help explore new ideas and identify those specific resources needed to implement them. Likewise, they would have access to continued feedback to adjust their proactive endeavor in order to be successful [51]. As a result, they would be more motivated to expand efforts in initiating change-oriented actions aimed at improving the work context. In contrast, when tasks are simpler, proactive behaviors are less required for achieving performance goals [68]. Further, less extensive support is required from supervisors for assisting employees with the demands of jobs with low complexity. For example, Scott and Bruce [69] found that LMX was more predictive of innovative behavior in complex jobs (e.g., engineering and scientists vs. technicians). In sum, strong, rather than weak, levels of job complexity would make the work situation congruent with LMX, thereby optimizing the motivational power of this social resource in enhancing proactive performance. Accordingly, we hypothesize the following (Hypothesis 3):

**Hypothesis** **3**  *Job complexity will moderate the positive relationship between LMX and proactive performance such that this relationship will be stronger (vs. weaker) when job complexity is higher (vs. lower).*

**Task Interdependence as a Moderator of TMX**. As argued by Srivastava and Singh [56], “the importance of team-member exchange quality is greater in work situations in which success is contingent upon strong social exchange relationships between team members” [p. 2]. On one hand, perceived embeddedness in a group has been identified as a boundary condition of the collective wellbeing–performance relationship [70]. Further, Chiaburu and Harrison’s meta-analysis [71] indicated that the correlation between co-worker support and performance is stronger when the job is socially intense (i.e., extent to which cooperation among co-workers is required). One way to capture this phenomenon is via task interdependence. Task interdependence refers to the degree to which an employee must share resources (e.g., material, information, or expertise) with coworkers to achieve expected performance [72]. High levels of interdependence mean more opportunities to collaborate with and influence others and to facilitate the performance of others in the group. 

TMX implies that an individual has access to supportive resources from coworkers (e.g., information, emotional support). However, support from coworkers may not always be relevant for bringing improvements in job performance, particularly if coworkers do not sufficiently understand the work context and the job mission of the focal employee. This could lead to suboptimal strategies to tackle the challenge at hand that may distract the employee with thoughts that are not useful for pursuing proactive efforts. Further, as discussed earlier, these supportive resources can sometimes come with disadvantages (e.g., social forces limiting change-oriented behaviors) that undermine proactive performance. 

We propose that high levels of task interdependence can make the resources offered by coworkers (e.g., TMX) more relevant to proactively implement new ideas at work. In jobs with interdependent tasks, employees would benefit more from coworkers’ support to implement their ideas because coworkers may use their own experience and knowledge to provide useful and contextualized resources to assist others. Further, in a highly interdependent environment, coworkers would develop a cohesion around the mission and tasks to perform rather than staying focused on the maintenance of supportive relationships. In the context of interdependent tasks, collaboration and cohesion within a group (i.e., the motivating potential of TMX) would lead to more initiatives and creative performance [19]. In contrast, in jobs with low task interdependence, TMX may be less instrumental to performance outcomes as coworkers are less familiar with each other’s work. Being less knowledgeable about others’ jobs, these coworkers could offer advice that is not applicable or relevant for the employee pursuing proactive efforts. Finally, the development of supportive relationships in the context of a low task interdependence could orient group members towards meeting individuals’ emotional needs. In such situations, a high TMX could be associated with some restrictive forces toward changes to preserve the group’s supportive role. Thus, TMX would not be favorable to proactive behaviors in a context of low task interdependence. In sum, high TMX is more likely to manifest its motivational potential for enhancing proactive behavior among employees with task-interdependent jobs. Therefore, we hypothesize the following:

**Hypothesis** **4**  *Task interdependence will moderate the positive relationship between TMX and proactive performance such that this relationship will be stronger (vs. weaker) when TMX is higher (vs. lower).*

### 1.3. Overall Moderated Mediation Model

So far, we have predicted that psychological wellbeing relates positively to proactive performance via LMX and TMX (Hypotheses 1 and 2). In addition, we have proposed that job complexity (Hypothesis 3) and task interdependence (Hypothesis 4) moderate the LMX–proactive performance and TMX–proactive performance relationships, respectively. Combining the mediating roles of LMX and TMX and the moderating roles of job complexity and task interdependence results in a moderated mediation model [73,74]. The indirect relationships between psychological wellbeing and proactive performance via LMX and TMX should be stronger when job complexity and task interdependence are higher. Wellbeing would foster LMX, which would be more instrumental to support proactive behaviors when workers have complex jobs. Likewise, wellbeing would enable TMX, which would be more instrumental for engaging in proactive endeavors when tasks are interdependent. These predictions are summarized in the following hypotheses (Hypotheses 5 and 6).

**Hypothesis** **5**  *Job complexity will moderate the positive indirect relationship between psychological wellbeing and proactive performance via LMX such that this indirect relationship will be stronger (vs. weaker) when job complexity is higher (vs. lower).*

**Hypothesis** **6**  *Task interdependence will moderate the positive indirect relationship between psychological wellbeing and proactive performance via TMX such that this indirect relationship will be stronger (vs. weaker) when task interdependence is higher (vs. lower).*

## 2. Materials and Methods

### 2.1. Participants and Procedure

Participants were recruited from 16 organizations located in the Quebec province, Canada. Cold calls were made to organizations listed in the provincial directory of knowledge-based organizations and to secondary references following these calls until we reached the target of a thousand potential participants for our multi-wave research project. Organizations’ representatives were contacted by the researchers and agreed to participate in a research project on employee wellbeing, performance and innovation. Organizations were operating in a variety of industries, including engineering, architecture, insurance, legal services, human resources, and aeronautics. The study involved three waves of data collection between 2015 and 2018. A time-lagged design was used where psychological wellbeing and control variables were measured at Time 1, LMX and TMX were measured at Time 2, and proactive performance was measured at Time 3 through supervisor reports. A time lag of three months between measurements was used, which provided enough time for temporal effects to be observed between wellbeing and the mediators, and between the mediators and proactive performance. The overall time span of six months can be considered optimal for detecting relationships between attitudes and behavior, while effects tend to decline over longer periods of time [75,76]. All measures were collected during work hours with online questionnaires using a Secure Sockets Layer protocol (hosted by Survey Monkey). Participants were presented with the study objectives, the ethical guidelines, and means available to obtain additional information. After providing their consent to participate, they accessed the survey through a personalized link provided in the email invitation. This link allowed the identification of the participants’ names and matching data across the three measurement waves. The project received approval from the University’s ethical committee (CERAS-2015-16-054-D).

At Time 1, 1038 employees participated in the survey, of which 941 provided complete responses. At Time 2, we contacted employees that had participated in the first-wave survey. Of these, 615 provided usable responses and did not change supervisors. These employees did not differ from those who participated only at Time 1 on wellbeing (t (939) = −0.52, ns). Further, they were not different on demographic characteristics (sex, age, education, tenure). At Time 3, supervisors rated employees’ proactive performance through online questionnaires containing the names of the employees to be assessed. Supervisors’ ratings were obtained for a total of 318 employees. These ratings were then matched by the research team with the responses obtained from employees. When comparing the final sample to the initial pool of respondents, they did not differ on wellbeing (t (939) = −1.23, ns), LMX (t (939) = −1.85, ns), and TMX (t (939) = −0.03, ns). Further, they were not different on demographic characteristics (sex, age, education, tenure). Therefore, attrition in participation should not represent a threat affecting the study results. In the final sample, most participants were women (55%), were aged between 25 and 45 years (64%), had a university degree (58%), and reported a tenure of more than 5 years (52%).

### 2.2. Measures

**Psychological wellbeing at work.** Wellbeing was measured with Gilbert et al.’s instrument [1]. This questionnaire is composed of 25 items measuring serenity, social harmony, and feelings of engagement at work (e.g., “I feel good, at peace with myself;” “I got along well with my colleagues;” “I found my work exciting and I wanted to enjoy every moment of it”). Participants were asked to indicate the extent to which they had experienced each wellbeing item in the previous month at work (1 = almost never; 5 = almost always). Previous studies indicated that the psychological wellbeing construct can be represented as a global, second-order factor [77]. Therefore, we averaged scores on items across the three components to create a global score of psychological wellbeing. Previous studies found global wellbeing to be reliable (αs = 0.91 and 0.92) [77,78]. 

**Job complexity.** Morgeson and Humphrey’s scale [67] was used to measure job complexity. Employees answered three items from a French version of this scale [79] to indicate their perception of job complexity (e.g., “The tasks on the job are simple and uncomplicated”—reverse coded). Previous studies indicated that this scale is reliable (α = 0.88) [79]. 

**Task interdependence.** The 5-item scale developed by Aubé et al. [80] was used to measure task interdependence (e.g., “I have to work closely with my colleagues to do my work properly”). Previous studies have indicated that this scale is reliable (α = 0.91) [81].

**LMX.** LMX was measured with the French version [82] of Graen and Uhl-Bien’s instrument [37]. This 7-item scale measures the extent to which the employee perceives having high quality exchanges with the supervisor. This scale includes items targeting employee contribution (e.g., “I have enough confidence in my supervisor that I would defend and justify his/her decision if he/she were not present to do so”) and supervisor contribution (e.g., “How well does your supervisor recognize your potential?”). Participants answered items using a 5-point scale (1 = rarely/not a bit/not at all/none/strongly disagree/extremely ineffective; 5 = very often/a great deal/full/very high/strongly agree/extremely effective). Previous studies have reported good reliability for this scale (α = 0.92) [82]. 

**TMX.** TMX was measured with Seers et al.’s scale [38]. A translation-back-translation procedure was performed to create a French version of this measure. This 10-item scale measures the extent to which employees perceive having high quality exchanges with coworkers. This scale targets employee contribution (e.g., “In busy situations, how often do you volunteer your efforts to help your colleagues”) and coworkers’ contribution (e.g., “How well do your colleagues recognize your potential?”). Participants answered items using the same 5-point scale as for LMX. Previous studies have indicated that this scale is composed of two highly correlated dimensions that can be merged to create an overall score [38,83,84]. Therefore, we computed a global score by averaging across all items. Reliability for this scale (α = 0.84) was similar to reliabilities reported in previous studies (α = 0.81–0.84) [38,83,84]. 

**Proactive performance.** The 3-item French version [85] of Griffin et al.’s scale [11] was used to measure proactive performance in core tasks. Supervisors assessed whether employees displayed proactive behaviors in their core tasks (e.g., “Comes up with ideas to improve the way in which your core tasks are done”). Supervisors indicated the extent to which (1 = totally disagree; 5 = totally agree) the employee had performed the behaviors in the past three months. Prior studies reported good reliability for this scale (αs = 0.91–0.94) [11,85].

**Control variables.** We controlled for age, education level, and organizational tenure as people with more work experience and knowledge (resulting from higher education, tenure, or age) may engage in more innovative thinking to find proactive solutions to work problems [86]. We also controlled for gender since men might be advantaged in obtaining resources for proactive behaviors as organizations tend to be more favorable to men than to women in providing resources [87]. Furthermore, we included a measure of self-reported proactive performance at Time 1 (3 items; α = 0.78) [85] to control for the potential effects of proactive behaviors on the quality of social interactions [88]. The inclusion of this variable as a control also helped account for the potential impact of proactive dispositions on exchange relationships [43]. Finally, we controlled for job autonomy (9 items; α = 0.86) [67] as a potential moderator of the relationship between LMX and proactive performance (see Appendix B).

## 3. Results

### 3.1. Confirmatory Factor Analysis

Prior to testing the hypotheses, we conducted confirmatory factor analyses (CFAs) with Mplus 7.11 [89] to assess the discriminant validity of our variables. For TMX (i.e., a two-dimensional construct), psychological wellbeing (i.e., a three-dimensional construct) and job autonomy (i.e., a three-dimensional construct comprising work scheduling autonomy, decision-making autonomy, and work method autonomy), the scores on the corresponding dimensions were used as indicators of their latent construct to ensure an adequate sample-size-to-parameter ratio. As can be seen in Table 1, the hypothesized eight-factor model (self-reported proactive performance, job autonomy, psychological wellbeing, job complexity, task interdependence, LMX, TMX, and proactive performance) displayed a good fit (χ^2^_[349]_ = 417.87, *p* < 0.01, comparative fit index (CFI) = 0.94, root mean square error of approximation (RMSEA) = 0.05, standardized root mean square residual (SRMR) = 0.05) and outperformed any simpler representations of the data (*p* < 0.01). Accordingly, these findings support the distinctiveness of the study variables (see also Appendix C). Descriptive statistics and correlations are presented in Table 2.

### 3.2. Hypotheses Testing

Because employees were nested in 16 organizations, we performed a series of hierarchical linear modeling (HLM) analyses using HLM 6.02 software [90] to test our hypotheses. We first ran a null model to determine if there was significant between-organization variance in LMX, TXM and proactive performance. Results indicated significant between-organization variance in LMX, (χ^2^ = 46.50, df = 15, *p* < 0.01, intra-class correlation [ICC]_[1]_ = 0.09), TMX (χ^2^ = 38.68, df = 15, *p* < 0.01, [ICC]_[1]_ = 0.07), and proactive performance (χ^2^ = 47.39, df = 15, *p* < 0.01, [ICC]_[1]_ = 0.09), thereby justifying the use of HLM. All study variables were group-mean centered as our model implied individual-level predictors and interactions between individual-level variables [91]. As recommended by Preacher and Selig [92], we used the Monte Carlo method to calculate confidence intervals for the hypothesized indirect and conditional indirect effects.

Table 3 presents the results of HLM analyses predicting LMX, TMX, and proactive performance, and provides the basic information for testing Hypotheses 1–6. Hypotheses 1 and 2 predicted that psychological wellbeing would be indirectly and positively related to proactive performance via LMX and TMX, respectively. Table 3 shows that wellbeing was positively associated with LMX (γ = 0.59, *p* < 0.01; Model 2) and TMX (γ = 0.49, *p* < 0.01; Model 5); in turn, LMX (γ = 0.19, *p* < 0.01), but not TMX (γ = −0.04, ns), was positively related to proactive performance (Model 8). Based on 20,000 Monte Carlo replications, the results revealed that the indirect effect of wellbeing on proactive performance via LMX was significant (0.11, 95% CI = 0.02, 0.21). Therefore, Hypothesis 1 is supported while Hypothesis 2 is not.

**Figure 2:**
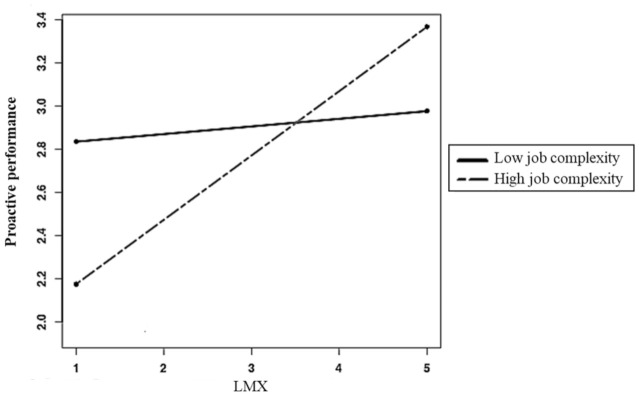
Proactive performance as a function of LMX at ±1 standard deviation of job complexity.

**Figure 3:**
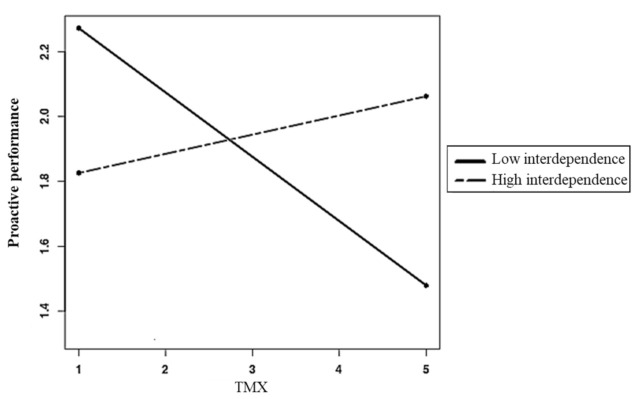
Proactive performance as a function of TMX at ±1 standard deviation of task interdependence.

Hypotheses 3 and 4 stated that TMX and LMX would be more strongly related to proactive performance at high levels of job complexity and task interdependence, respectively. As shown in Table 3 (Model 9), the LMX x job complexity interaction (γ = 0.09, *p* < 0.05) and the TMX x task interdependence interaction (γ = 0.11, *p* < 0.05) were significant. A likelihood ratio test [93] indicated that the model including these interaction terms (Model 9) yielded a better fit (Δχ2 = 12.61, df = 4, *p* < 0.01) than the model with no interaction terms (Model 8). Simple slopes analyses [94] revealed that the LMX–proactive performance relationship was significantly positive (γ = 0.30, *p* < 0.01) at high levels (i.e., 1 SD above the mean) of job complexity but non-significant (γ = 0.03, ns) at low levels (i.e., 1 SD above the mean) of it (Figure 2), thus supporting Hypothesis 3. Further, the TMX–proactive performance relationship was significantly negative when task interdependence was low (γ = −0.20, *p* < 0.05) but non-significant when it was high (γ = 0.06, ns) (Figure 3). This pattern is not entirely consistent with Hypothesis 4 as the latter predicted the relationship between TMX and proactive performance to be stronger and positive when task interdependence was high.

Finally, to test whether job complexity (Hypothesis 5) and task interdependence (Hypothesis 6) moderated the indirect relationship between psychological wellbeing and proactive performance via LMX and TMX, respectively, we used 20,000 Monte Carlo replications of the data to generate 95% bias-corrected CIs for the indirect effects of wellbeing at different values of these moderators. As predicted, the indirect effect of psychological wellbeing on proactive performance via LMX was significantly positive when job complexity was high (0.18, CI = 0.08, 0.29) but non-significant when it was low (0.02, CI = −0.07, 0.11). Hypothesis 5 is thus supported. The indirect effect of psychological wellbeing via TMX was significantly negative when task interdependence was low (–0.10, CI = −0.19, −0.01) but non-significant when it was high (0.03, CI = −0.06, 0.13). As for Hypothesis 4, this result is not entirely supportive of Hypothesis 6.

### 3.3. Supplemental Analyses

We performed a series of supplemental analyses to examine several alternative effects, namely whether (a) LMX and TMX interacted to predict proactive performance, (b) job autonomy or (c) task interdependence moderated the relationship between LMX and proactive performance, (d) job complexity moderated the relationship between TMX and proactive performance, and (e) job complexity and task interdependence moderated the relationship between wellbeing and LMX and TMX (i.e., the first stage of the mediation).

First, as TMX has been reported in previous research to moderate the relationship between LMX and work performance [41], we examined the interaction between LMX and TMX predicting proactive performance. Model 10 (Table 3) indicates that the LMX × TMX interaction was unrelated to proactive performance (γ = −0.10, ns), while the LMX × job complexity (γ = 0.09, *p* < 0.01) and the TMX × task interdependence (γ = 0.12, *p* < 0.05) interactions remained significant. Second, as prior research found job autonomy to moderate the relation between LMX and change-related behaviors [65], we tested whether it would moderate the LMX–proactive performance relation. Model 11 (Table 3) showed that the LMX × job autonomy interaction was unrelated to proactive performance (γ = −0.03, ns), while the LMX × job complexity remained significant (γ = 0.09, *p* < 0.01). Third, we tested whether task interdependence moderated the relationship between LMX and proactive performance and whether job complexity moderated the relationship between TMX and proactive performance. Model 12 (Table 3) indicates that task interdependence did not moderate the LMX–proactive performance relation (γ = 0.03, ns) and that job complexity did not moderate the TMX–proactive performance relation (γ = −0.05, ns), while the LMX × job complexity (γ = 0.09, *p* < 0.01) and TMX × task interdependence (γ = 0.11, *p* < 0.05) interactions remained significantly related to proactive performance. Fourth, we examined whether job complexity and task interdependence moderated the first stage of the mediation sequence. The results showed that job complexity did not moderate the wellbeing–LMX (γ = 0.04, ns; Table 3, Model 3) and wellbeing–TMX (γ = 0.02, ns; Table 3, Model 6) relations. Likewise, task interdependence did not moderate the relation between wellbeing and LMX (γ = −0.02, ns; Table 3, Model 3) and TMX (γ = 0.04, ns; Table 3, Model 6).

## 4. Discussion

The goal of this study was to investigate how and under what conditions psychological wellbeing can contribute to proactive performance. Based on the COR theory [25], we posited that employees with higher levels of wellbeing may develop more positive exchange relationships with their supervisor (LMX) and coworkers (TMX), two social resources that can be instrumental to proactive performance. Analyses revealed that LMX, but not TMX, mediated the wellbeing–proactive performance relation. While the idea that wellbeing fuels TMX was supported, TMX was unrelated to proactive performance. Further, we explored whether the motivational potential of LMX and TMX was moderated by specific job characteristics [62]. As expected, LMX was more strongly related to proactive performance when job complexity was higher. While we expected that the contribution of TMX to proactive performance would be stronger when task interdependence is high, the results indicated that TMX negatively contributed to proactive performance when task interdependence was low. Taken together, our study indicates that psychological wellbeing is indirectly related to proactive performance through two social exchange mechanisms, both of which were subject to boundary conditions. The theoretical and practical implications of these findings are discussed in the next sections.

### 4.1. Theoretical Implications

This research has implications for how we theorize psychological wellbeing’s role in the workplace as well as for the understanding of the social mechanisms through which it leads to proactive performance. Based on our results, future theorizing of these relationships ought to go beyond the simplistic paradigm of the happy-productive worker [96,97]. Based on the COR theory, we proposed that psychological wellbeing would facilitate the development of general social resources relevant for work adjustment. Consistent with the motivational fit theory [24], we also contended that these resources could become more (vs. less) relevant to the enactment of proactive behaviors depending on job requirements [20]. Our study has the potential to resolve the inconsistent findings concerning the relation between employee wellbeing and performance [9] as well as those regarding the association between social resources and proactive performance [20].

Our findings supported our assumption that psychological wellbeing eases the access to social resources at work. However, the development of the two general resources examined (i.e., LMX and TMX) does not provide an equally relevant set of resources for employees to engage in proactive behaviors in their jobs. The results indicate that LMX is conducive to proactive behaviors while exchange relationships with coworkers are not. This suggests that supervisors can provide more instrumental resources for job improvement (e.g., organizational resources, coaching, and feedback) and more socio-political support than coworkers, who are recognized as major information and emotional support providers [48]. This result is compatible with the finding that instrumental support from a supervisor is more important than parallel support from coworkers for job performance [98]. This also fits the proactivity literature where LMX was found to be more strongly related to proactive performance than TMX across various job conditions [20]. Our study adds to this stream of research by revealing that LMX is a more instrumental resource in the context of complex tasks, which are typically completed by knowledge workers. Presumably, knowledge workers benefit more from LMX because it allows the relationship with the supervisor to be customized to their needs [51,52].

One reason why TMX was unrelated to proactive performance might be that proactivity was targeted at employees’ core tasks. Maybe a different pattern of results would emerge if proactive behaviors were targeting the team [11,99]. We found that TMX was detrimental to proactivity when task interdependence was low. In line with the motivational fit perspective [24], one may speculate that TMX would be beneficial for proactivity directed at the team level in the context of high task interdependence. Presumably, the “collective” motivational potential of TMX would be facilitated as a driver of team-directed proactivity when employees must coordinate work efforts with coworkers. Another possibility is that proactive attempts to challenge the status quo could threaten the high-quality relationships with coworkers in task-interdependent situations [23]. For example, De Dreu and West [100] found that task interdependence was negatively related to minority dissent, a type of proactive behavior that implies voicing personal views against the majority [101]. However, conflicts about tasks resulting from working interdependently with others can also stimulate change-oriented behaviors when they coexist with a positive mood or with an innovative group climate [10]. In such conditions, group cohesiveness might enhance, rather than attenuate, the effects of psychological wellbeing on proactive performance [102]. These contrasting perspectives suggest that the ability of task interdependence to indirectly enhance the relationship between employee wellbeing and proactive performance through TMX may be dependent on additional boundary conditions (e.g., the ability to deal with conflicts that can arise in a work group).

The current study also has theoretical implications for research on the “energy-to” factors related to proactivity [12]. Prior research has suggested that activated positive emotions directly foster proactive behaviors at work [50,103]. However, employee wellbeing at work does not boil down to highly activated positive emotions. In this study, we used a measure of psychological wellbeing which is aligned with the employees’ phenomenological experience of being psychologically fulfilled at their job. Such experience implies having a positive view of oneself, the social context, and job activities [1,2]. As such, feeling well at work may not only have a direct effect on proactive behaviors but can exert an indirect effect by facilitating the development of other general resources that can be beneficial for responding to unpredictable opportunities for improvements in one’s job. Rather than being contradictory, these two approaches are complementary. In line with the COR theory, the indirect perspective, by emphasizing the role of general resources for work adjustment, can be complemented by the direct perspective, which is theoretically anchored in the principle of energy activation [104]. Interestingly, our moderated mediation findings concur with this integrative possibility: job complexity played an activation role by providing a proximal “reason-to” display proactive behaviors to employees in high-quality LMX relationships. Further theoretical integrations related to the “energy-to” reasons for proactivity could help clarify how the distal, proximal and boundary factors jointly explain the emergence of proactive performance.

### 4.2. Managerial Implications

This study has practical implications for the promotion of proactive work performance. Our results indicated that the positive relationship between psychological wellbeing and proactive performance occurs because wellbeing relates to quality exchange relationships with supervisors. Therefore, human resource management interventions aimed at nurturing employees’ wellbeing may boost the availability of social resources (i.e., LMX), which is conducive to proactive performance. Psychological wellbeing and LMX will contribute the most to employee proactivity when the task is complex. Therefore, human resource managers should see the promotion of wellbeing and LMX relationships as a ground out of which proactive behaviors can flourish. As such, promoting psychological wellbeing and LMX should be diligently encouraged and be viewed as complimentary means that can enhance performance. As a boundary condition, we nevertheless must caution that the promotion of psychological wellbeing does not appear a relevant strategy to improve employees’ proactivity when their tasks are simple.

Further, this study discloses a possible adverse effect of psychological wellbeing in relation to proactive performance, namely when this effect occurs through social exchange relationships with coworkers in the context of low-interdependent tasks. When task interdependence is low, intense social exchanges with coworkers should be avoided to prevent drops in proactive performance. In sum, our results suggest that TMX does not add value over LMX in stimulating proactive performance. Therefore, augmenting the quality of relationships with supervisors remains the best means to improve proactive performance. Interestingly, recent research on followership indicates that employees can play an active role in crafting relationships with their leaders [105].

Moreover, by providing evidence for the positive effect of job complexity on the indirect relationship between wellbeing and proactive performance via LMX, our study emphasizes the importance of increasing job complexity among psychologically healthy employees who have high-quality relationships with their supervisors in order to bring out their proactive potential. To this end, organizations could broaden the tasks assigned to such employees and increase their decision-making authority. Likewise, it is advisable to make jobs mentally stimulating and challenging, and to allow employees to use cognitive skills as well as to exploit opportunities for learning, exploring, and experimenting. However, it is worth noting that, in the case of jobs that are already inherently complex, such as knowledge-based jobs, further increasing the levels of their complexity (e.g., by augmenting information processing or task variety) might result in increased job overload, which may reduce the resources needed to exert proactive efforts. Accordingly, the management of job complexity should be based on a preliminary diagnosis of current job characteristics to optimize the effectiveness of the aforementioned job design practices in spurring proactive performance among psychologically healthy employees who benefit from satisfactory LMX. Supporting these recommendations, and consistent with our study findings, Volmer and colleagues [65] showed that LMX was most beneficial to employee innovative performance (i.e., a form of proactive behavior) when employees experienced high levels of job autonomy (i.e., a core characteristic of knowledge jobs).

Overall, our results suggest that improving psychological health is a good starting point for developing constructive social exchange relationships and fostering proactive performance in jobs characterized by high-complexity and low interdependence. However, the knowledge-based characteristics of our study sample set key boundaries to the application of this general practical recommendation, suggesting that the well-being-oriented route to effective social exchanges and proactive performance might be more fruitful when applied to knowledge workers, namely to those high-skilled employees who use theoretical and analytical knowledge acquired through formal education in creating new and useful business solutions.

### 4.3. Strengths, Limitations, and Directions for Future Research

This research has strengths and limitations that need to be discussed. The first strength was that a three-wave design was used to examine our moderated mediation model. Such design allowed us to accurately assess the processes as they unfold in our theorized model [106]. As such, the measurement of wellbeing at Time 1, social resources at Time 2, and proactive performance at Time 3 followed recommended practices [106,107]. The three-month interval between measurement times was selected based on prior research suggesting that attitudes-to-behavior relationships tend to diminish after six months [75,76,108]. However, our design does not allow us to draw conclusions in terms of causality. For example, a time-lagged design is less powerful than a cross-lagged panel design where directional relationships between variables can be examined and reverse effects can be tested. However, we controlled for self-reported proactive performance at Time 1, which helped alleviate concerns regarding potential effects of proactive performance on LMX and TMX.

For establishing appropriate time lags between measurements, while short time lags (i.e., weeks or a few months) appear advisable [109], the relative stability of phenomena under study should be considered for defining optimal time intervals. For example, previous research has suggested that the link between employee wellbeing and positive social exchange behaviors can develop quickly (daily to three months) [54,110,111,112]. However, social resources such as LMX and TMX may require longer periods to develop and stabilize before they can influence proactive behaviors. As such, the three-month lag between psychological wellbeing and social resources was appropriate. In contrast, the relationship between social resources and proactive performance may become stronger over periods of time longer than three months [113]. Future research could explore this issue by measuring proactivity multiple times (e.g., over six months or one year). Proactive behaviors in core tasks [77] and performance ratings in general [114] are often more stable than is generally thought. Thus, allowing for more time between measurement occasions for these outcomes would be well-advised.

The second strength of this study was the use of supervisor ratings to assess proactive performance. As a formal agent of the organization, the supervisor is the legitimate individual who can provide this assessment [115]. Further, other-ratings reduce the social desirability of performance assessments and common method variance bias in the attitude-performance relationship [108]. However, there are also some limitations to this approach. Liking effects may bias the supervisor’s judgment of subordinate performance. Following Dulebohn et al. [116], there is a strong relationship between supervisor liking and the assessment of employee extra-role behaviors. Therefore, one may wonder if the LMX-proactive performance relation is partly affected by a liking bias. Still, Dulebohn et al. [116] found that LMX accounted for a larger proportion of variance in extra-role performance than liking.

Future studies aiming at examining the role of psychological wellbeing in proactive performance may explore some worthwhile avenues. First, future research may want to investigate this relationship by focusing on a collective target of proactive performance (i.e., team-directed proactive performance) or adopting a group-level perspective [10]. This may extend what we currently know of the relationship between wellbeing and proactive performance and would have benefits for team-based organizations. Second, it would be interesting to examine whether cultural dimensions (e.g., collectivism vs. individualism) moderate the relationship between LMX and proactive performance, as observed in previous research [41]. In relation to this point, it should be noted that our study was conducted in Canada, a country characterized by a horizontal-individualism configuration [117]. In this type of society, people focus on their individuality to define themselves and tend to value equal relationships with figures of authority. This cultural feature could have influenced how the individuals surveyed in this study construed their representation of being interdependent to others or how they weighted their individual (vs. collective) interests in the enactment of proactive performance. Therefore, the generalization of our results to collective-vertical societies, such as those located in Asia, as well as in highly multicultural contexts, is uncertain. Accordingly, future research is warranted to investigate whether collectivist values may act as another boundary condition in the relationship between TMX and proactive performance. Third, this study focused on social exchange relationships as intervening factors between psychological wellbeing and proactive performance relationships. Other relevant mechanisms may be motivation [118] and cognitive flexibility [119], which may have their own boundary conditions [120].

## 5. Conclusions

The present study explored two pathways through which psychological wellbeing may affect proactive performance, namely social exchange relationships with supervisors (LMX) and coworkers (TMX). While findings indicated that LMX was the unique mediator, the relationship between both LMX and TMX and proactive performance was moderated by job characteristics, namely job complexity and task interdependence. We hope future research will further explore how and when psychological wellbeing contributes to work performance.

## Figures and Tables

**Table 1:** Confirmatory Factor Analysis Results: Fit Indices.

Model	*χ* ^2^	*df*	Δ*χ*^2^	Δ*df*	CFI	RMSEA	SRMR
Hypothesized eight-factor model	417.87 *	349	–	–	0.94	0.05	0.05
Six-factor models							
Combining job complexity and task interdependence	1367.85 *	356	949.98 *	7	0.78	0.09	0.11
Combining job complexity and job autonomy	1154.59 *	356	736.72 *	7	0.83	0.08	0.09
Combining task interdependence and job autonomy	1109.36 *	356	691.49 *	7	0.84	0.08	0.07
Combining LMX and TMX	855.67 *	356	437.80 *	7	0.89	0.07	0.06
One-factor model	3629.37 *	377	3211.50 *	28	0.31	0.16	0.15

**Note:***N* = 318. CFI = comparative fit index; RMSEA = root mean square error of approximation; SRMR = standardized root mean square residual; LMX = leader-member exchange; TMX = team-member exchange. * *p* < 0.01.

**Table 2:** Descriptive Statistics and Correlations.

Variable	*M*	*SD*	1	2	3	4	5	6	7	8	9	10	11	12
1. Gender	1.45	0.50	–											
2. Age	2.97	1.08	−0.13 *	–										
3. Educational level	3.70	0.94	0.21 **	−0.07	–									
4. Organizational tenure	4.53	1.18	−0.08	0.46 **	−0.05	–								
5. Psychological wellbeing	4.17	0.44	−0.04	0.01	−0.12 *	−0.14 **	(0.92)							
6. Job complexity	4.55	1.51	0.09	0.12 *	0.27 **	0.20 **	−0.12 **	(0.92)						
7. Task interdependence	5.33	1.16	0.15 **	−0.14 *	0.27 **	−0.06	0.08	0.13 *	(0.85)					
8. Job autonomy	5.30	1.12	0.25 **	−0.03	0.25 **	0.02	0.20 **	0.18 **	0.27 **	(0.86)				
9. SRPP	4.09	0.61	−0.07	−0.02	−0.06	−0.14 *	0.42 **	–0.03	0.07	0.17 **	(0.78)			
10. LMX	3.71	0.73	−0.01	−0.05	−0.11	−0.10	0.42 **	−0.08	0.16 **	0.21 **	0.18 **	(0.87)		
11. TMX	3.45	0.62	0.09	−0.07	0.07	0.03	0.39 **	−0.09	0.29 **	0.11	0.25 **	0.39 **	(0.84)	
12. Proactive performance	3.75	0.77	−0.01	−0.13 *	−0.06	−0.15 **	0.23 **	−0.01	0.07	0.07	0.10	0.25 **	0.08	(0.86)

**Note:** N = 318. SRPP = self-reported proactive performance; LMX = leader-member exchange; TMX = team-member exchange. For Gender: 1 = female, 2 = male. For Age: 1 = ≤ 25 years, 2 = 26–35 years, 3 = 36–45 years, 4 = 46–55 years, 5 = 56–65 years, 6 = ≥ 66 years. For Educational level: 1 = primary school, 2 = secondary school, 3 = college, 4 = undergraduate, 5 = graduate. For Organizational tenure: 1 =< 6 months, 2 = 6 months-1 year, 3 = 1–2 years, 4 = 2–5 years, 5 = 5–10 years, 6 = 10–15 years, 7 = >15 years. * *p* < 0.05; ** *p* < 0.01.

**Table 3:** Hierarchical Linear Modelling Results for LMX, TMX and Proactive Performance.

Variables	LMX			TMX			Proactive Performance					
	Model 1	Model 2	Model 3	Model 4	Model 5	Model 6	Model 7	Model 8	Model 9	Model 10	Model 11	Model 12
Gender	−0.01	−0.01	−0.00	0.05	0.05	0.06	0.13	0.13	0.14	0.14	0.13	0.14
Age	0.03	0.01	0.00	−0.03	−0.05	−0.05	−0.09 *	−0.11*	−0.10 *	−0.10 *	−0.10 *	−0.10
Educational level	−0.06	−0.03	–0.07	0.06	0.08	0.04	−0.05	−0.03	−0.07	−0.07	−0.08	−0.07
Organizational tenure	0.00	0.01	0.00	0.06 **	0.07 **	0.07 **	−0.02	−0.02	−0.02	−0.03	−0.03	−0.02
SRPP	0.17 **	−0.00	−0.01	0.27 **	0.12 *	0.12 *	0.05	−0.02	−0.05	−0.04	−0.05	−0.05
PWB		0.59 **	0.56 **		0.49 **	0.44 **		0.21 *	0.20 *	0.18 *	0.18	0.21 *
LMX								0.19 **	−0.23 **	−0.22	−0.38 *	−0.26
TMX								−0.04	−0.66 *	−0.74 *	−0.67 *	−0.64 *
Job complexity			0.02			–0.03			−0.31 **	−0.30 **	−0.34 *	−0.33 **
Task interdependence			0.09 *			0.13 **			−0.30	−0.33	−0.32	−0.29
Job autonomy											0.03	
LMX × Job complexity									0.09 *	0.09 *	0.09 *	0.09 **
TMX × Task Interdependence									0.11 *	0.12 *	0.11 *	0.11 *
LMX × TMX										−0.10		
LMX × Job autonomy											−0.03	
PWB × Job complexity			0.04			0.02						
PWB × Task interdependence			−0.02			0.04						
LMX × Task interdependence												0.03
TMX × Job complexity												−0.05
*R* ^2^	0.03	0.12	0.13	0.09	0.11	0.16	0.04	0.08	0.12	0.12	0.12	0.12
Δ*χ*^2^(*df*)		35.10(1) **	7.76(4)		36.13(1) **	20.86(4) **		17.16(3) **	12.61(4) **	1.45(1)	1.25(2)	1.95(2)
Deviance	688.51	653.41	645.65	561.56	525.43	504.57	710.62	693.46	680.85	679.40	679.59	678.89

**Note.** N = 318. Models 3, 6, and 10–12 refer to supplemental analyses. *R^2^* values are calculated based on proportional reduction in error variance resulting from predictors in the models of Table 3 [95]. The fit of Models 10–12 is compared to the fit of Model 9. PWB = Psychological wellbeing; SRPP = Self-reported proactive performance; LMX = leader-member exchange; TMX = team-member exchange. For Gender: 1 = female, 2 = male. For Age: 1 = ≤ 25 years, 2 = 26–35 years, 3 = 36–45 years, 4 = 46–55 years, 5 = 56–65 years, 6 = ≥ 66 years. For Educational level: 1 = primary school, 2 = secondary school, 3 = college, 4 = undergraduate, 5 = graduate. For Organizational tenure: 1 = < 6 months, 2 = 6 months-1 year, 3 = 1–2 years, 4 = 2–5 years, 5 = 5–10 years, 6 = 10–15 years, 7 = >15 years. * *p* < 0.05; ** *p* < 0.01.

## Data Availability

The data presented in this study are available on request from the corresponding author.

## Appendix A

Following review studies on LMX [38,39] and TMX [43], as well as a range of studies targeting both constructs [121,122,123,124,125], these variables are defined and measured in this study at an individual level, with follower rather than leader perceptions.

## Appendix B

Based on theory [126] and research [127] on proactivity, self-reported proactive performance can be considered the behavioral manifestation of proactive personality, since this latter represents “a stable disposition to take personal initiative in a broad range of activities and situations” (p. 847) [128], which is the core characteristic of proactive behaviors.

## Appendix C

Because one of the dimensions of well-being, namely social harmony, has an interpersonal focus, it could present conceptual redundancies with the interpersonal variables of LMX and TMX. We therefore explored the bivariate correlations and compared different CFA models to determine the distinctiveness between social harmony, and LMX and TMX. The bivariate correlations of social harmony with LMX (r = 0.36, *p* < 0.01) and TMX (r = 0.37, *p* < 0.01) were below 0.50, thus representing medium-strength correlations [129]. The CFA results revealed that a three-factor model encompassing social harmony, TMX and LMX was a significantly better fit to the data than either a two-factor model merging social harmony and LMX (Δ*χ^2^* [2] = 351.18, *p* < 0.01) or a two-factor model merging social harmony and TMX (Δ*χ^2^* [2] = 342.66, *p* < 0.01). Taken together, the results from the correlation and CFA analyses indicate that the construct of social harmony is distinct from and not overlapping with the constructs of LMX and TMX.
